# Neurological soft signs and social cognition in patients with schizophrenia: the missing link

**DOI:** 10.1192/j.eurpsy.2026.10525

**Published:** 2026-07-31

**Authors:** K. Guevara Mihalev, G. Onchev

**Affiliations:** 1 Division of Adult Mental Health Care, Stavanger University Hospital, Stavanger, Norway; 2 Psychiatry, Medical Univeristy Sofia, Sofia, Bulgaria

## Abstract

**Introduction:**

Schizophrenia is a debilitating mental disorder characterised by a pronounced decline in mental, physical, and social functioning.

**Objectives:**

Our study aims to investigate the relationship between the socio-cognitive and the subtle motor and sensory symptoms of schizophrenia, which are, respectively, represented by facial emotion recognition deficits (FERD) and neurological soft signs (NSS).

**Methods:**

Thus, we assessed 60 patients diagnosed with schizophrenia. FERD was measured with the “Averaged Karolinska Directed Emotional Faces” set, and NSS were assessed with the Neurological Evaluation Scale (NES).

**Results:**

A strong and significant negative correlation was found between the NES total score and the FERD total score (r=−0.7, p <0.001). There was also a significant negative correlation between several of the subscores of both instruments.

**Conclusions:**

The results show that impaired social cognition is significantly associated with soft neurological disturbances in patients suffering from schizophrenia. Taken together, this reinforces the notion that schizophrenia is characterised by dysfunction and disconnection of a complex cerebral network, which can probably be influencedby psychosocial and physical non pharmacological therapeutic interventions.

**Disclosure of Interest:**

None Declared

